# Clinical Outcomes of Deep Transverse Friction Massage and Active Exercises on Pain, Quality of Life, and Maximum Mouth Opening in Patients With Temporomandibular Disorders: A Case Series

**DOI:** 10.7759/cureus.112159

**Published:** 2026-07-06

**Authors:** Shashank Kumar, Ranganathan Arunmozhi, Sumit Khattri

**Affiliations:** 1 School of Physiotherapy and Allied Health Sciences, Sardar Bhagwan Singh University, Dehradun, IND; 2 Department of Psychiatry, Shri Guru Ram Rai University, Dehradun, IND

**Keywords:** active exercise, physiotherapy treatment, quality of life, temporomandibular joint dysfunction, ultrasound therapy

## Abstract

Temporomandibular joint (TMJ) disorder refers to a cluster of conditions characterized by pain in the TMJ or its surrounding tissues, functional limitations of the mandible, or clicking in the TMJ during motion. The TMJ is a bilateral synovial joint on both sides of the craniomandibular complex, involved in chewing, swallowing, speech, and other automatic movements such as yawning, grinding, or clenching. A total of 10 patients with TMJ dysfunction were included in this case series, and all were diagnosed and referred by a dentist. Physical examination was carried out by the therapist to check for muscle spasm, restricted range of motion if available, clicking sound in mouth opening or closing, intensity of pain, and quality of life using the Oral Health Impact Profile-14 (OHIP-14) questionnaire scale and the Jaw Functional Limitation Scale (JFLS) questionnaire. Deep transverse friction massage (DTFM) and conventional therapy were given to all patients for three sessions per week for six weeks. The combined approach of DTFM and conventional physiotherapy may effectively reduce pain, improve jaw function, increase mouth opening, and enhance quality of life in patients with TMJ dysfunction.

## Introduction

The temporomandibular joint (TMJ) is a synovial joint located bilaterally within the craniomandibular complex and plays a vital role in mastication, deglutition, speech, and other parafunctional activities. Various pathophysiological, behavioral, psychological, and structural factors may alter normal masticatory function and contribute to the development of temporomandibular disorders (TMDs) [1,2]. The International Association for the Study of Pain defines TMD as pain in the muscles of mastication associated with restricted jaw movement and joint sounds [3].

TMD is also characterized by clicking or popping sounds during jaw movement, headache, earache, and facial pain. In some cases, hypertrophy of the masticatory muscles and abnormal tooth wear due to parafunctional habits such as bruxism may also be observed [4]. A recent systematic review reported a global prevalence of TMD ranging from 31% to 34% [5]. In India, a study reported a prevalence of 53.2% among individuals aged 16-60 years using the Fonseca questionnaire, with males more commonly affected [6].

As TMD is multifactorial in nature, conservative management is considered the first-line treatment approach, including physiotherapy, pharmacological management, occlusal splints, psychotherapy, and self-care strategies. Physiotherapy interventions commonly include manual therapy, therapeutic exercises, relaxation techniques, and electrotherapy modalities [7,8].

Deep transverse friction massage (DTFM) is a manual therapy technique widely used in the management of myofascial pain syndromes. It helps improve tissue mobility, restore muscle function, and reduce adhesions through repetitive transverse movements applied perpendicular to muscle fibers [9-11]. Although DTFM has demonstrated effectiveness in various musculoskeletal conditions, limited evidence is available regarding its role in TMD. Therefore, the present study aimed to investigate the effect of conventional physiotherapy combined with DTFM on pain, range of motion, quality of life, and disability in individuals with TMD.

## Materials and methods

Study design

A prospective case series was conducted among 10 patients diagnosed with TMD. Data were collected prospectively from Jams Dental Clinic and Tara Dental Clinic. Patients were referred to physiotherapy following a dental diagnosis of TMD. Ethical approval for the study was obtained from Sardar Bhagwan Singh University. Outcome measures were assessed at baseline, at the end of the third week, and after completion of the sixth week of treatment.

Inclusion criteria

The inclusion criteria were a heterogeneous population aged 18 to 50; myogenic TMD, including masticatory muscle spasm; history of peri-auricular pain of at least three months' duration, with or without joint sounds; and referral from a dentist with a diagnosis of TMJ dysfunction.

Exclusion criteria

The exclusion criteria included TMD with structural changes in the TMJ due to known arthritis; known rheumatoid disease; diagnosed ankylosing spondylitis, disc degeneration or Bell’s palsy; history of malignancy in the last five years; other physical contraindications, such as fracture, dislocations, known metabolic diseases; known hematologic disorders (such as anemia or leukemia); patients could not have symptoms arising from active pathology of the head, face, and jaw.

Intervention

Participants received DTFM combined with conventional physiotherapy, three sessions per week for six weeks. The conventional physiotherapy protocol comprised therapeutic ultrasound, administered at an intensity of 1.2 W/cm^2^ for 10 minutes per session using an HMS ultrasound unit (HMS Medical Systems, Chennai, India) operating at a frequency of 1 MHz with a coupling agent. Active range of motion exercises for the jaw were also prescribed as part of the treatment protocol that included unresisted mouth opening and closing with a five-second hold at the end range for 10 repetitions and three sets. DTFM was administered for 10-15 minutes per session. The targeted muscles included the masseter, temporalis, and medial pterygoid muscles. During the intervention, patients were positioned in side-lying, and friction massage was applied perpendicular to the muscle fibers using the pad of the index finger supported by the middle finger. This technique was applied with firm, rhythmic transverse strokes directly to the identified tender areas within each muscle, gradually increasing pressure to tolerance. As per the patient's comfort, a few seconds of rest were also given to minimize discomfort during applying DTFM.

Treatment fidelity

The intervention was administered by the primary investigator, a physiotherapist currently pursuing a PhD in physiotherapy (overall 10 years of experience). All treatment procedures were conducted under the supervision of a senior physiotherapist with more than 15 years of clinical and academic experience in musculoskeletal rehabilitation. Prior to participant recruitment, the intervention protocol was standardized and followed consistently throughout the study. The duration, sequence, and dosage of DTFM, therapeutic ultrasound, and active jaw exercises were maintained uniformly across all participants to ensure treatment fidelity. The participant’s attendance was recorded at each treatment session, and verbal feedback was obtained regularly to ensure compliance with the prescribed exercise program.

Outcome measures

Pain Intensity Using the Numeric Pain Rating Scale (NPRS)

Pain intensity was assessed using the NPRS, an 11-point scale ranging from 0 (no pain) to 10 (worst imaginable pain). The intraclass correlation coefficient of the NRS is 0.95 [12].

Jaw Functional Limitation Scale (JFLS)

Functional limitation of the jaw was evaluated using the JFLS. This assessment contains 20 questions, each of which is scored 0 (no limitation) to 10 (severe limitation), yielding a maximum total score of 200. The reliability coefficient of the JFLS is 0.82 to 0.99 [13].

Oral Health Impact Profile-14 (OHIP-14)

Oral health-related quality of life was assessed using the OHIP-14. A validated 14-item questionnaire encompasses seven domains: functional limitation, physical pain, psychological discomfort, physical disability, psychological disability, social disability, and handicap. The intra-class correlation coefficient of the OHIP-14 scale is 0.84 [14,15].

Digital Vernier Caliper

It is a device used to measure linear size. The width of round and cylindrical-shaped things is also measured by this scale; it is done by positioning the caliper’s jaws on both sides of the border. The intraclass correlation coefficient value of the vernier caliper scale exceeded 0.75 for the digital calipers [16].

Ethical consideration

Written informed consent was obtained from all participants before participation. The study protocol was approved by the Institutional Review Board (IRB) of Sardar Bhagwan Singh University and conducted in accordance with the Declaration of Helsinki.

Statistical analysis

Data analysis was performed using Microsoft Excel (Microsoft Corp., Redmond, WA, USA). Due to the descriptive nature of the study, only descriptive statistics, including mean, median, and standard deviation, were calculated.

## Results

Participants

The present case series evaluated the effect of intervention over a period of six weeks using outcome measures, the NPRS, the JFLS, the OHIP-14 questionnaire, and vertical mouth opening using a vernier caliper. All variables showed improvement; however, OHIP-14 showed significant improvement among all variables, indicating substantial improvement in patients’ oral health-related quality of life. The JFLS showed progressive improvement over the period of six weeks of treatment sessions. Whereas the vernier caliper showed gradual improvement in mouth opening, indicating less improvement among all variables.

Table 1 shows that the mean age of participants was 33.50 ± 7.40 years, indicating that most participants were young to middle-aged adults. For the NPRS, the mean score reduced from 7.40 ± 1.35 during the first week to 5.30 ± 1.25 in the third week and further to 2.90 ± 1.10 by the sixth week. This demonstrated a considerable reduction in pain intensity over time. The JFLS also showed marked improvement. The mean score decreased from 41.70 ± 11.08 at baseline to 29.70 ± 8.61 in the third week and 16.65 ± 9.76 in the sixth week, indicating improvement in jaw function and reduction in functional limitation.

**Table 1:** Descriptive Statistics of Demographic Characteristics and Outcome Measures at Baseline, Week 3, and Week 6 (n = 10) Vertical mouth opening was measured using a digital vernier caliper (VC) in millimeters (mm). NPRS: Numeric Pain Rating Scale; JFLS: Jaw Functional Limitation Scale; OHIP-14: Oral Health Impact Profile-14; SD: standard deviation

Variables	Mean	Median	Standard Deviation
Age	33.5	33	7.4
Group	1	1	0
NPRS First Week	7.4	8	1.35
NPRS Third Week	5.3	6	1.25
NPRS Sixth Week	2.9	3	1.1
JFLS First Week	41.7	40	11.08
JFLS Third Week	29.7	28.5	8.61
JFLS Sixth Week	16.65	12.5	9.76
OHIP First Week	69.27	71.42	15.43
OHIP Third Week	45	46.42	12.17
OHIP Sixth Week	16.38	14.28	10.68
Vernier First Week	31.36	31.93	2.99
Vernier Third Week	32.97	33.43	2.1
Vernier Sixth Week	34.9	35.8	2.19

Similarly, the OHIP-14 scores declined from 69.27 ± 15.43 at the first week to 45.00 ± 12.17 at the third week and 16.38 ± 10.68 at the sixth week. This suggests considerable improvement in oral health-related quality of life following treatment. In contrast, the vernier caliper measurements (mouth opening) increased progressively from 31.36 ± 2.99 mm at baseline, 32.97 ± 2.10 mm in the third week, and 34.90 ± 2.19 mm in the sixth week, indicating enhancement in vertical mouth opening. The median values were close to the mean values for most variables, suggesting relatively symmetrical data distribution without major outliers. The standard deviation values were moderate across variables, indicating acceptable variability among participants and relatively consistent treatment response.

This descriptive analysis demonstrates clinically meaningful improvement in pain, jaw function, oral health-related quality of life, and mouth opening from the first week to the sixth week. These findings indicate that the intervention protocol used in the case series was effective in improving outcomes in a patient with TMJ disorders.

## Discussion

The findings of the study demonstrated progressive improvement in all outcome measurements over the six-week intervention period. The pain intensity showed a consistent decrease in the pain intensity from baseline to the sixth week. This improvement may be attributed to the combined effect of DTFM, active jaw exercises, and ultrasound therapy. Manual therapy techniques are known to stimulate neurophysiological mechanisms that help in pain reduction, reduction of muscle spasm, and improvement of local circulation [2]. DTFM also enhances collagen fiber extensibility and soft tissue mobility, thereby reducing discomfort and improving the functional movement of the TMJ. It encourages increased circulation, decreases muscle spasm, and increases extensibility of the collagen fibers [17]. The improved elasticity and extensibility of the muscles, ligaments, and tissues surrounding the TMJ allow smoother and more comfortable jaw movements [18].

Patients also reported better jaw mobility and reduced difficulty during functional activities such as chewing and mouth opening. These finding associated with improved muscle flexibility, reduced soft tissue tightness, and enhanced coordination of masticatory muscles following physiotherapy intervention. Active jaw exercises possibly contributed to the restoration of normal movement patterns and functional stability of the TMJ [19].

The observed improvements are consistent with the literature supporting the role of conservative physiotherapy intervention in the management of TMDs. Manual techniques may contribute to pain modulation through neurophysiological mechanisms, while therapeutic exercises may improve muscle coordination and mandibular mobility. Nevertheless, because multiple interventions were administered simultaneously, the individual contribution of DTFM cannot be isolated from the effect of active exercise and therapeutic ultrasound. Therefore, the observed outcomes should be interpreted as the result of a multimodal physiotherapy approach rather than a single treatment component.

TMD commonly affects physical comfort, emotional well-being, eating, communication, and daily activities. Reduction in pain and increased functional disability observed in the study may have contributed to decreased psychological stress and improved oral health-related quality of life. The findings suggest that physiotherapy intervention positively influences both physical and psychological aspects of TMD.

The combined intervention approach may have facilitated smoother mandibular movement and improved TMJ mobility. Ultrasound therapy was used as an adjunct modality on the affected area for 10 minutes at 1.2 W/cm^2^ per session to reduce pain and inflammation. The thermal and non-thermal effects of therapeutic ultrasound may enhance blood circulation, tissue metabolism, and soft tissue healing, which could further support pain reduction and functional improvement [19].

Overall, the findings of this case series suggest that DTFM combined with conventional physiotherapy is more beneficial in reducing pain, improving jaw function, enhancing mouth opening, and improving quality of life in patients with TMD. These findings should be interpreted within the context of the case series study design, which does not include a control group or statistical inference testing. A randomized controlled trial with a larger sample size is recommended to establish the effectiveness of this treatment approach and to examine its relative superiority to other intervention strategies.

Limitations

Several limitations should be considered when interpreting the findings of this series. First, the sample size was small (n = 10), limiting the statistical power and generalizability of the result. Second, the absence of a control or comparison group prevents the determination of whether the observed improvements were attributable to the intervention, natural recovery, placebo effects, or other factors. Third, participants were recruited through referrals from dental clinics, which may introduce selection bias. Fourth, no long-term follow-up assessment was conducted; therefore, the sustainability of the treatment remains unknown. Future studies employing randomized controlled designs, larger sample sizes, and extended follow-up periods are recommended. Finally, a visual illustration of the DTFM technique was not included in the manuscript. Although a detailed textual description procedure has been provided, inclusion of photographs or schematic diagrams may further improve reproducibility and educational value in future publications.

## Conclusions

This case series observed improvements in pain intensity, jaw functional limitation, oral health-related quality of life, and vertical mouth opening among participants with TMDs who received DTFM combined with active jaw exercise and therapeutic ultrasound over six weeks. While these findings suggest that the intervention may be associated with positive clinical outcomes, the absence of a control group and a small sample size preclude any conclusion regarding treatment effectiveness or causality. Further randomized controlled trials with large samples and long-term follow-up are warranted to determine the efficacy of this intervention approach.
